# Effect of Inflammatory Mediators Lipopolysaccharide and Lipoteichoic Acid on Iron Metabolism of Differentiated SH-SY5Y Cells Alters in the Presence of BV-2 Microglia

**DOI:** 10.3390/ijms20010017

**Published:** 2018-12-20

**Authors:** Edina Pandur, Edit Varga, Kitti Tamási, Ramóna Pap, Judit Nagy, Katalin Sipos

**Affiliations:** 1 Department of Pharmaceutical Biology, Faculty of Pharmacy, University of Pécs, Rókus u. 2., H-7624 Pécs, Hungary; edina.pandur@aok.pte.hu (E.P.); edit.varga@aok.pte.hu (E.V.); kitti.tamasi@aok.pte.hu (K.T.); pap.ramona@pte.hu (R.P.); 2 Department of Anaesthesiology and Intensive Therapy, Medical School, University of Pécs, Ifjúság út 13., H-7624 Pécs, Hungary; nagy.judit@pte.hu

**Keywords:** inflammation, iron metabolism, neuron, microglia, bilaminar co-culture

## Abstract

Lipopolysaccharide (LPS) and lipoteichoic acid (LTA), the Gram-negative and the Gram-positive bacterial cell wall components are important mediators of neuroinflammation in sepsis. LPS and LTA are potent activators of microglial cells which induce the production of various pro-inflammatory cytokines. It has been demonstrated that disturbance of iron homeostasis of the brain is one of the underlying causes of neuronal cell death but the mechanisms contributing to this process are still questionable. In the present study, we established monocultures of differentiated SH-SY5Y cells and co-cultures of differentiated SH-SY5Y cells and BV-2 microglia as neuronal model systems to selectively examine the effect of inflammatory mediators LPS and LTA on iron homeostasis of SH-SY5Y cells both in mono- and co-cultures. We monitored the IL-6 and TNFα secretions of the treated cells and determined the mRNA and protein levels of iron importers (transferrin receptor-1 and divalent metal transporter-1), and iron storing genes (ferritin heavy chain and mitochondrial ferritin). Moreover, we examined the relation between hepcidin secretion and intracellular iron content. Our data revealed that LPS and LTA triggered distinct responses in SH-SY5Y cells by differently changing the expressions of iron uptake, as well as cytosolic and mitochondrial iron storage proteins. Moreover, they increased the total iron contents of the cells but at different rates. The presence of BV-2 microglial cells influenced the reactions of SH-SY5Y cells on both LPS and LTA treatments: iron uptake and iron storage, as well as the neuronal cytokine production have been modulated. Our results demonstrate that BV-2 cells alter the iron metabolism of SH-SY5Y cells, they contribute to the iron accumulation of SH-SY5Y cells by manipulating the effects of LTA and LPS proving that microglia are important regulators of neuronal iron metabolism at neuroinflammation.

## 1. Introduction

Sepsis is among the most common causes of morbidity and mortality worldwide [1]. In sepsis, the central nervous system (CNS) is one of the first organs that is affected [2]. Sepsis is associated with production of pro-inflammatory cytokines, impaired brain microcirculation, and disturbed neurotransmitter secretion [3]. Cytokine production is one of the underlying cause of neuroinflammation leading to disruption of blood–brain barrier, neuronal oxidative stress, and microglial activation [4,5]. Microglia are the resident immune cells of the brain responsible for the production of cytokines (tumor necrosis factor α (TNFα), interleukin-1β (IL-1β), and interleukin-6 (IL-6)), neurotrophic factors, nitrogen oxide (NO), and reactive oxygen species that lead to neuronal cell death [6,7,8]. They also play a crucial role in the alteration of blood–brain barrier and blood–cerebrospinal fluid barrier in systemic inflammation [9].

Lipopolysaccharide (LPS) and lipoteichoic acid (LTA), the two lipid-linked polymers of bacterial cell wall components, are found in Gram-negative and Gram-positive bacteria, respectively [10,11]. LTA evokes the induction of several inflammatory mediators in different cell types (e.g., macrophages, lung cells, liver cells, and glial cells) including TNFα, IL-1β, IL-6, IL-8, monocyte chemoattractant protein-1 (MCP-1), macrophage inflammatory proteins (MIP-1a and MIP-2), myeloperoxidase, and NO [12,13]. LPS mainly initiates the production of pro-inflammatory cytokines like TNFα, IL-1β, IL-6, and NO in both glial cells and neurons [14,15]. It has been demonstrated that LPS and LTA induce the production of reactive oxygen species (ROS) and activate NFκB (nuclear factor kappa B) pathway which leads further production of pro-inflammatory cytokines [16].

LPS and LTA are recognized by Toll-like receptors (TLRs) which are involved in the innate immune system [17,18]. Upon ligand binding TLRs dimerize and initiate myeloid differentiation primary response gene 88 (MyD88)-dependent or independent pathways leading to the production of IL-6, IL-1, and TNFα [19]. TLR2 recognizes lipoproteins and other components of Gram-positive bacterial cells, such as LTA [20]. TLR4 recognizes LPS, a structural component of the outer membrane of Gram-negative bacterial cells, double-stranded RNA or flagellin and various endogenous ligands and necrotic cells [17,21,22].

The presence of TLRs has been demonstrated both in the CNS and in neural cell cultures. Evidences prove that microglia and neurons both express TLR4 and TLR2 [23]. TLR signaling in the CNS strongly influences the neuroinflammatory responses [24,25].

The presence of iron is critical in the normal development of brain functions. It is needed for myelin synthesis and for neurotransmitter production [26]. Iron uptake in the neuronal cells occurs mostly via the well described transferrin–transferrin receptor (TfR) system. Other mechanisms for iron import in different cell types of the CNS involve divalent metal transporter-1 (DMT-1), brain melanotransferrin, or lactoferrin [27]. The main iron storage protein in the brain is ferritin [28]. Mitochondrial ferritin (FTMT), a ferritin-like protein has been proved to act as a modulator of cellular iron homeostasis. FTMT is expressed predominantly in neurons and sequesters free iron protecting the cell from oxidative damage [29]. Neurons and microglial cells have the capacity to accumulate and store large quantities of iron, although microglial cells are more efficient in this process than neurons [30]. Iron accumulation leads to oxidative damage, mitochondrial dysfunction and neuronal cell death [31].

The systemic iron metabolism and homeostasis are dependent on the proper regulation of hepcidin, a small antimicrobial peptide which is mainly expressed in the liver [32]. Hepcidin is translated as preprohepcidin, from which the 24 amino acids long N-terminal presequence is cleaved in the endoplasmic reticulum. The prohepcidin is transported into the Golgi apparatus where the furin proprotein convertase cuts it into the 25 amino acids long mature hepcidin [32]. Under conditions of iron overload in order to limit iron export, hepcidin binds to its receptor ferroportin, and triggers its internalization and intracellular degradation [33]. Iron homeostasis is also controlled by the iron regulatory proteins (IRPs) which bind iron responsive elements (IREs) of regulated messenger RNAs [34]. Inflammatory conditions are the major regulators of hepcidin synthesis and therefore the overall iron homeostasis. It has been described that inflammatory stimuli (IL-6, TNFα, and LPS treatments) of the CNS cells lead to perturbation of iron homeostasis [35,36].

LPS is a common inflammatory mediator to induce inflammatory processes (e.g., pro-inflammatory cytokine production) in neurons and microglia. Meanwhile, the role of Gram-positive bacterial cell wall component LTA has not been investigated in the context of neuroinflammation and iron metabolism.

In the present study we established bilaminar co-culture systems of differentiated SH-SY5Y cells and BV-2 cells which are relevant cell models to investigate neuroinflammatory processes [8,37,38]. We utilized these models to describe new implications of iron metabolism in neuroinflammation. We evaluated the effects of inflammatory mediators LPS and LTA on the iron uptake and storage in differentiated SH-SY5Y cells. Microglia are known to be able to modulate neuronal functions and they have an important role in neuroinflammatory processes. Therefore we investigated the alterations of the iron metabolism of differentiated SH-SY5Y cells in the presence of BV-2 microglia to reveal the modifications mediated by these immune cells.

## 2. Results

### 2.1. Determination of Toxicity of LPS and LTA on SH-SY5Y

**Figure 1:**
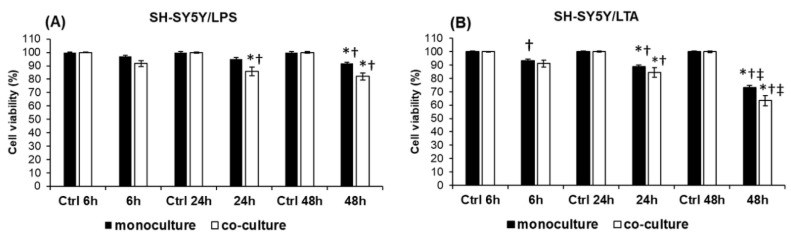
Viability of mono- and co-cultured SH-SY5Y cells after LPS and LTA treatments. Cell viability was determined using Cell Counting Kit-8 (Sigma-Aldrich Kft., Budapest, Hungary) and was expressed as percentage of the control cells. (**A**) Viability of LPS treated SH-SY5Y cells and co-cultured SH-SY5Y cells. (**B**) Viability of LTA treated SH-SY5Y cells and co-cultured SH-SY5Y cells. The columns represent mean values and error bars represent standard errors of the mean (SEM) of four independent determinations. Asterisk stands for *p* < 0.01 between mono- and co-cultures. Double cross means *p* < 0.01 between LPS and LTA treatments. Cross shows *p* < 0.01 compared to the untreated controls.

Our preliminary results on LTA- and LPS-treated SH-SY5Y cells revealed that 1 µg of bacterial cell wall component per ml of culture media is the optimal working concentration to induce inflammatory processes. These conclusions were based on mRNA expression experiments. Next, we determined the optimal time intervals of LPS and LTA administrations with cell viability assays. Lipopolysaccharide decreased cell viability only to 92% after 48 h while lipoteichoic acid decreased viability to 73% at 48 h treatment in monocultures. In the presence of BV-2 cells the viability of SH-SY5Y cells decreased to 82% at 48 h after LPS treatment and to 67% at 48 h after LTA treatment (Figure 1).

### 2.2. LPS and LTA Have Distinct Effects on the mRNA Expressions of the Iron Uptake and Storage Genes in SH-SY5Y Cells

Our primary goal was to reveal the effects of BV-2 cells on the iron metabolism of SH-SY5Y cells in the separate treatments with LPS or LTA, but our results also demonstrated that the two different bacterial cell wall components triggered altered responses in monocultured SH-SY5Y cells.

**Figure 2:**
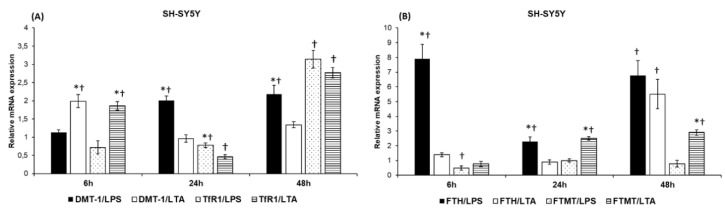
Effects of LPS and LTA treatments on the mRNA expressions of iron uptake and iron storage genes in SH-SY5Y cells. Real-time PCR was performed with the SYBR green protocol using gene-specific primers. β-actin was used as a housekeeping gene for the normalization and relative expression of controls was considered as 1. The mRNA expressions of the treated cells were compared to their appropriate controls (6 h, 24 h, or 48 h). (**A**) mRNA expression levels of DMT-1 and TfR1 of LPS- and LTA-treated SH-SY5Y cells. (**B**) mRNA expression levels of FTH and FTMT of LPS-and LTA-treated SH-SY5Y cells. The columns represent mean values and error bars represent standard errors of the mean (SEM) of three independent determinations. Asterisk indicates *p* < 0.01 between LPS and LTA treatments. Cross marks indicate *p* < 0.01 compared to the untreated controls.

The mRNA analysis demonstrated that iron uptake genes (DMT-1 and TfR1) showed different expression levels in SH-SY5Y cells in the presence of LPS and LTA. DMT-1 expression levels were significantly elevated at 24 h and 48 h in the presence of LPS, while LTA treatment increased its level significantly as early as 6 h, although the mRNA expression of DMT-1 was downregulated to the control level at 24 h (Figure 2A). TfR1 showed a different expression profile as well: it was elevated at 6 h and 48 h in case of LTA treatment while the LPS treatment significantly increased the TfR1 mRNA levels only at 48 h (Figure 2A). These results may suggest that SH-SY5Y cells react later to LPS treatment due to its different action, and both DMT-1 and TfR1 contribute to LPS-mediated iron uptake. In the case of LTA treatment, DMT-1 levels begin to change earlier (6 h) and at late stage of the treatment the increasing expression of TfR1 may take the place of DMT-1 in iron uptake.

The distinct effects of LPS and LTA treatments are more obvious in case of iron storage genes. The mRNA expressions of FTH were elevated at each time points of LPS treatment but with different altitudes (Figure 2B). Meanwhile LTA treated cells showed increased FTH expression only at 48 h. FTMT mRNA levels were increased in case of LTA treatment of SH-SY5Y cells, while LPS did not seem to affect significantly FTMT mRNA expression (Figure 2B). These results presume that LPS acts mainly on FTH expression while LTA affects primarily FTMT mRNA level. The results also suggest that LPS acts on cytosolic iron stores while LTA modifies both the mitochondrial and cytosolic iron stores.

### 2.3. LPS and LTA Act Differently on the Hepcidin Secretion and Iron Content of the SH-SY5Y Cells

**Figure 3:**
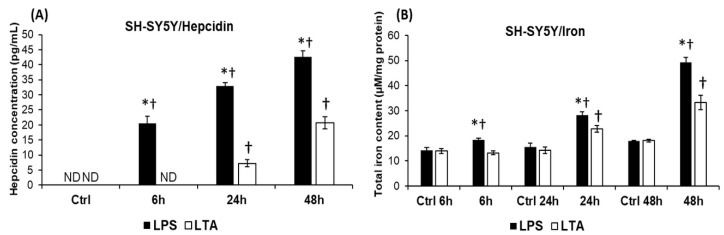
Effects of LPS and LTA treatments on hepcidin secretion and on iron content of SH-SY5Y cells. Hepcidin content of cell culture supernatants was determined with Hepcidin 25 bioactive ELISA Kit. The hepcidin contents of the control media did not change during the experiments therefore only one control was represented in Figure A. Intracellular iron contents of SH-SY5Y cells were determined using a colorimetric ferrozine-based assay. Iron contents of the untreated control cells were measured the same way and at the same time points of the experiments (6 h, 24 h, and 48 h). (**A**) Hepcidin concentrations in the cell culture media of the LPS and LTA treated SH-SY5Y monocultures. (**B**) Total iron contents of LPS and LTA treated SH-SY5Y cells. The columns represent mean values and error bars represent standard errors of the mean (SEM) of four independent determinations. ND stands for not detected. Asterisk marks *p* < 0.01 between LPS and LTA treatments. Cross marks *p* < 0.01 compared to the untreated controls.

Next, we determined the production of the major iron regulatory hormone hepcidin of LPS and LTA treated SH-SY5Y cells. Hepcidin secretions showed significant difference between the two treatments. In case of LPS treatment hepcidin secretion increased gradually from 6 h and this elevation was significantly higher than in case of LTA treatment (Figure 3A). LTA increased hepcidin production only in the late stages of the experiments—at 24 h and 48 h—and levels were significantly lower compared to LPS treatment (Figure 3A).

The iron contents of the treated SH-SY5Y cells followed the changes of hepcidin secretion. The higher hepcidin level resulted in higher intracellular iron content in case of LPS treated SH-SY5Y cells (Figure 3B). Increased iron levels were only measured at 24 h and 48 h in case of LTA treated cells but these values were significantly lower compared to the LPS treated cells (Figure 3B). These results suggest that hepcidin acts on SH-SY5Y cells with an autocrine fashion, and causes intracellular iron accumulation.

### 2.4. BV-2 Cells Alter the IL-6 and TNFα Cytokine Productions of SH-SY5Y Cells

**Figure 4:**
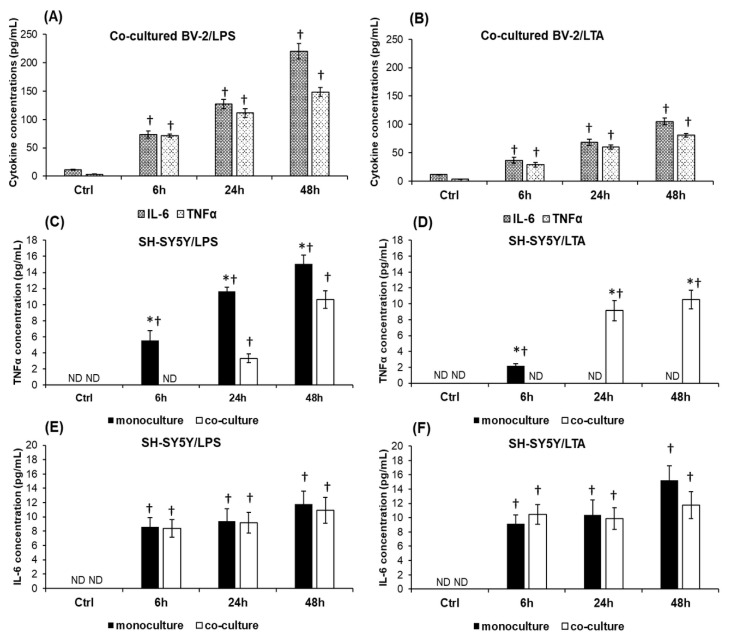
Determination of IL-6 and TNFα secretions of co-cultured BV-2 cells and mono- and co-cultured SH-SY5Y cells. Secreted IL-6 and TNFα concentrations of the culture media were determined with IL-6 and TNFα ELISA Kits specific for human or mouse cytokines. (**A**) IL-6 and TNFα productions of co-cultured BV-2 cells treated with LPS. (**B**) IL-6 and TNFα productions of co-cultured BV-2 cells treated with LTA. (**C**) IL-6 secretions of mono- and co-cultured LPS treated SH-SY5Y cells. (**D**) IL-6 secretions of mono- and co-cultured LTA treated SH-SY5Y cells. (**E**) TNFα secretion of mono- and co-cultured LPS treated SH-SY5Y cells. (**F**) TNFα production of mono- and co-cultured LTA treated SH-SY5Y cells. The columns represent mean values and error bars represent standard errors of the mean (SEM) of three independent determinations. The cytokine contents of the control media did not change during the experiments therefore only one control was represented in each figure. ND stands for not detected. Cross means *p* < 0.01 compared to the controls. Asterisk marks *p* < 0.01 between mono- and co-cultures.

Production of pro-inflammatory cytokines is a reliable marker of microglial activation. We investigated not only the microglial but the neuronal IL-6 and TNFα secretions as well, to reveal the reaction of SH-SY5Y cells to inflammatory mediators. The elevated levels of secreted cytokines prove that both cell types responded with IL-6 and TNFα production to the inflammatory mediators, LPS and LTA. In Figure 4A,B it can be seen that both bacterial cell wall components increased the secretion of microglial IL-6 and TNFα, although LTA exerted a 50% lower effect on the cells than LPS.

TNFα concentrations showed gradually elevated levels in the LPS treated monocultures of neuronal cells (Figure 4C). The presence of BV-2 cells in the LPS treated co-cultures caused a delay in the SH-SY5Y TNFα production and decreased the magnitude of the secretion (Figure 4C). LTA exerted different effect on the TNFα production of SH-SY5Y cells (detected only at 6 h) (Figure 4D) and BV-2 cells altered not only the time of secretion (delayed to 24 h) but they caused significant elevation of TNFα production compared to the monoculture (Figure 4D). The measured TNFα concentrations point out that LPS and LTA have different effect on TNFα secretion showing remarkable elevation only in case of LPS treatment, while in the co-cultures LPS has a lower effect on TNFα secretion and LTA is able to trigger TNFα production. In the SH-SY5Y monocultures the IL-6 protein level showed a moderate elevation during both treatments (8.47–11.76 pg/mL in the case of LPS and 9.14–15.21 pg/mL in the case of LTA), meanwhile BV-2 cells did not affect significantly the IL-6 production of neurons compared to the monoculture (Figure 4E,F).

### 2.5. Western Blot Analysis of the Iron Related Genes Reveals Alterations between the Mono- and Co-Cultured SH-SY5Y Cells

Inflammatory mediators together with other factors and conditions (e.g., iron regulatory proteins, microRNAs (miRNAs), and iron availability) are known to exert regulatory functions in the iron homeostasis of different cell types. We investigated whether the two different bacterial cell wall components influenced iron uptake and/or iron storage in SH-SY5Y cells and whether microglia, the immune cells of the brain were able to attenuate or enhance the effects of LPS and LTA on the iron metabolism of neuronal cells. DMT-1 and TfR1, which are responsible for iron uptake, and FTH and FTMT—iron storage protein encoding genes—were examined to reveal if there are any differences at protein expression levels.

**Figure 5:**
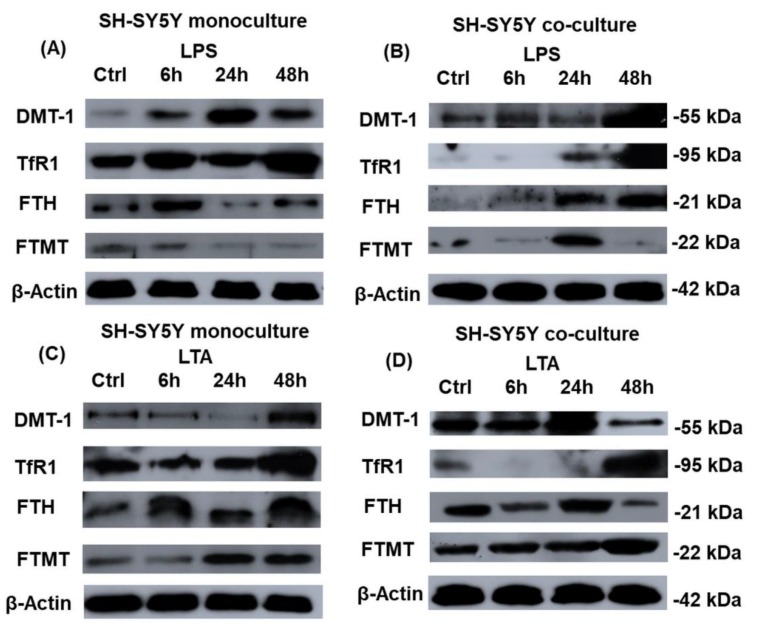
Western blot analyses of proteins involved in iron uptake and storage in mono- (**A**,**C**) and co-cultured (**B**,**D**) SH-SY5Y cells. Pelleted cells were lysed and their protein contents were measured. The same amount of protein (15 µg) from each sample was separated by SDS-PAGE using 12% or 14% polyacrylamide gels, transferred by electroblotting to nitrocellulose membranes and probed with anti-DMT-1, anti-TfR1, anti-FTH, and anti-FTMT polyclonal rabbit antibodies according to the manufacturers’ protocols. DMT-1 and TfR1 expressions reflect iron uptake capacity of SH-SY5Y cells. FTH and FTMT protein levels show the mechanisms of iron storage in SH-SY5Y cells. The experiments were repeated at least three times. Optical density analyses can be seen in Figure S1.

DMT-1 protein expression was elevated from 6 h to 48 h in the LPS treated monoculture. BV-2 cells retarded the expression of DMT-1 in the co-cultured SH-SY5Y cells, it was only increased at 48 h. We could detect also elevation of the expression of TfR1 in the LPS treated monoculture at 6 h and 48 h and in the presence of microglia, TfR1 protein levels were increased as well at 24 h and 48 h (Figure 5A,B) suggesting that the TfR1 level is regulated by LPS and is modified by BV-2 cells.

FTH levels were similar to the control level at 6 h and 48 h in monocultured, LPS-treated SH-SY5Y cells, but at 24 h remarkable down regulation occurred suggesting a negative feedback regulation or short term iron redistribution in the cells. Increasing FTH protein expression pattern was found in the LPS treated co-cultured SH-SY5Y cells suggesting that BV-2 cells notably affect cytosolic iron stores. FTMT protein levels were decreased at 24 h and 48 h in the LPS-treated monocultures. Interestingly, in the co-cultures, the FTMT protein level was increased at 24 h and then decreased suggesting that in the presence of microglia FTH may take the role of FTMT at 48 h in iron storage of SH-SY5Y cells (Figure 5A,B). This alteration may contribute to the protection of mitochondria from iron toxicity when mitochondrial iron store is filled.

In case of LTA treatments there was a decrease at 24 h of the DMT-1 protein in the monoculture which alteration did not appear in the co-culture suggesting an alteration in the translational regulation of this gene in the presence of BV-2 cells. TfR1 protein level showed elevated expression in the LTA treated monoculture suggesting that SH-SY5Y cells utilize TfR1 for iron import. In the co-cultured SH-SY5Y cells TfR1 began to be elevated only at 48 h suggesting that at earlier time points DMT-1 is used for iron uptake instead of TfR1, and then TfR1 takes the role or work together with DMT-1 (Figure 5C,D).

FTH protein levels were similar to the control level at the examined time points of LTA treatment of the monocultured SH-SY5Y cells. In the LTA treated co-culture, microglia seemed to decrease FTH protein levels of SH-SY5Y cells compared to the monocultures with an exception at 24 h when it increased. FTMT expression was increased at the late stages of the LTA treatments in both mono- and co-cultures. Based on these results it is revealed that both FTH and FTMT levels were elevated at 24 h and 48 h in the LTA treated monocultures. At the same time points BV-2 cells could decrease FTH and increase FTMT levels suggesting that FTMT may take the role of FTH (Figure 5C,D), which is in contrast with the results of LPS treated SH-SY5Y cells (Figure 5A,B).

### 2.6. BV-2 Cells Modify the Hepcidin Secretion of SH-SY5Y Cells

**Figure 6:**
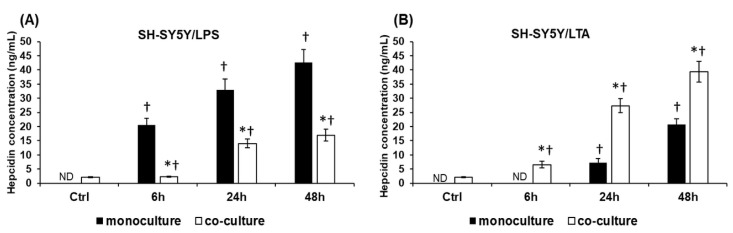
Determination of the concentration of secreted hepcidin from cell culture medium. The mature hepcidin content of each sample was determined with Hepcidin 25 bioactive ELISA Kit. (**A**) Hepcidin concentrations in the cell culture media of the LPS-treated SH-SY5Y monocultures and co-cultures. (**B**) Hepcidin productions of the LTA treated mono- and co-cultured SH-SY5Y cells measured from the culture medium. The columns represent mean values and error bars represent standard errors of the mean (SEM) of four independent determinations. The hepcidin contents of the control media did not change during the experiments therefore only one control was represented in the figures. Asterisk marks *p* < 0.01 between the mono- and co-cultures. Cross means *p* < 0.01 compared to the controls. ND means not detected.

Inflammation is one of the most important positive modulators of hepcidin expression, the major regulator of iron metabolism. To investigate the effects of BV-2 cells on hepcidin production of the LPS- and LTA-treated SH-SY5Y cells we determined hepcidin secretion of SH-SY5Y cells in mono- and co-cultures using competitive ELISA. The LPS treated SH-SY5Y cells showed increasing hepcidin levels with time (Figure 6A) in the monoculture. These changes were significantly higher than in the LPS treated co-cultured SH-SY5Y cells suggesting that BV-2 cells may inhibit hepcidin secretion and/or maturation. In the case of LTA treatment we revealed opposite results: the co-cultured SH-SY5Y cells secreted significantly higher amount of hepcidin compared to the monoculture (Figure 6B). The results suggest that the presence of BV-2 cells affects the hepcidin synthesis of SH-SY5Y cells: they suppress hepcidin secretion in case of LPS treatment while they trigger hepcidin production in case of LTA treatment.

### 2.7. Transcriptional Regulation of Hepcidin Antimicrobial Peptide (HAMP) Expression in the LPS- and LTA-Treated Mono- and Co-Cultured SH-SY5Y Cells

**Figure 7:**
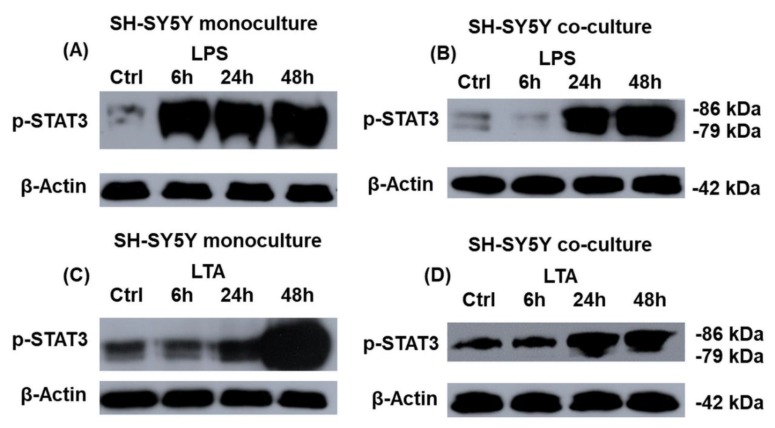
Western blot analyses of phospho-STAT3 in mono- (**A**,**C**) and co-cultured (**B**,**D**) SH-SY5Y cells. SH-SY5Y cells were collected and pelleted cells were lysed and their protein contents were measured. The same amount of protein (15 µg) from each sample was separated by SDS-PAGE using 12% polyacrylamide gel, transferred by electroblotting to nitrocellulose membranes and probed with anti-p-STAT3 polyclonal rabbit antibody according to the manufacturer’s protocol. p-STAT3 reflects the activation of JAK/STAT signaling pathway affecting HAMP transcription. The experiments were repeated at least three times. Optical density analyses can be found in Figure S2.

Preprohepcidin mRNA expression is regulated by STAT3 transcription factor, among other intracellular factors (e.g., SMAD transcription factors, HIF1α, and MAP kinase cascades). The STAT3 protein is phosphorylated by the IL-6 cytokine receptor/Janus kinase signaling pathway activated by inflammatory signals. Therefore, we examined STAT3 transcription factor phosphorylation in the monoculture and the co-cultured SH-SY5Y cells to reveal whether this process contributes to the increased hepcidin production in neurons. STAT3 phosphorylation was induced in the LPS and LTA treated monocultures (Figure 7A,C) with different rates. During LPS treatment, the STAT3 phosphorylation increased from 6 h, meanwhile LTA could initiate STAT3 phosphorylation at 24 h and after a remarkable increase occurred at 48 h, although there was no significant difference between IL-6 secretions of the treated cells. In the co-cultures STAT3 phosphorylation begun later, at 24 h in case of both treatments (Figure 7B,D), suggesting influencing role of BV-2 cells on cell signaling pathways in SH-SY5Y cells. Although these results strengthen the role of STAT3 phosphorylation in the regulation of hepcidin expression but other factors produced by BV-2 cells or by SH-SY5Y cells or the intracellular iron content may affect hepcidin production of SH-SY5Y cells as well.

### 2.8. Changes in the Expression of Iron Uptake Proteins and Hepcidin Contribute to the Increased Iron Content of SH-SY5Y Cells

**Figure 8:**
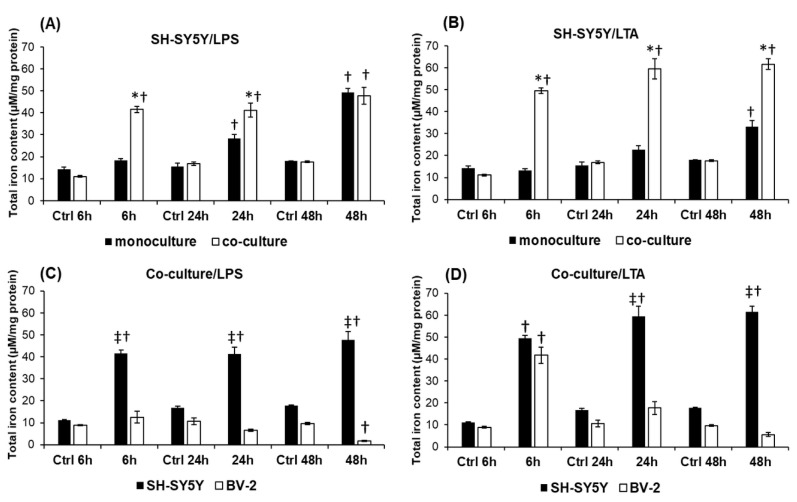
Comparisons of the total iron contents of mono- and co-cultured SH-SY5Y cells and co-cultured SH-SY5Y and BV-2 cells. Intracellular iron contents of SH-SY5Y and BV-2 cells were determined using a colorimetric ferrozine-based assay. Iron contents of the untreated control cells were measured the same way and at the same time points of the experiments (6 h, 24 h, and 48 h). (**A**) Total iron contents of LPS-treated mono- and co-cultured SH-SY5Y cells. (**B**) Total iron contents of the LTA-treated mono- and co-cultured SH-SY5Y cells. (**C**) Intracellular iron contents of SH-SY5Y cells and BV-2 cells in LPS-treated co-cultures. (**D**) Intracellular iron contents of SH-SY5Y cells and BV-2 cells in LTA treated co-cultures. The columns represent mean values and error bars represent standard errors of the mean (SEM) of four independent determinations. Asterisk stands for *p* < 0.01 between mono- and co-cultures. Double cross means *p* < 0.01 between SH-SY5Y cells and BV-2 cells. Cross means *p* < 0.01 compared to the controls.

To prove that changes in the expression of iron uptake and storage proteins show correlation with the variation of the intracellular iron content, we measured cellular total iron content using a ferrozine-based assay. Our results revealed that the iron content of the co-cultured SH-SY5Y cells was significantly higher in both treatments compared to the monocultures (Figure 8A,B)—except at 48 h of LPS treatment where there was no difference between the mono- and co-cultures (Figure 8A). The results suggest that BV-2 cells can increase the iron content of SH-SY5Y cells in the presence of LPS or LTA. Moreover, BV-2 cells alone, without any treatment, could increase neuronal iron content since the iron content of control co-cultured SH-SY5Y cells changed with time of cultivation (Figure 8C,D). Elevation of the iron content in the co-cultured SH-SY5Y cells raised the possibility that this extra iron was not imported exclusively from the culture medium but the BV-2 cells contributed directly to these changes as well. To reveal this hypothesis we measured the iron content of the BV-2 cells in the co-cultures. Increased microglial total iron content was measured only at 6 h of LTA treatment (Figure 8C). At 24 h and 48 h in both treatments, the BV-2 cells decreased their iron content meanwhile the total iron content of SH-SY5Y cells increased (Figure 8B,C). These data suggest the presence of iron transport between the two cell types in the co-cultures and the important role of microglia in iron accumulation of SH-SY5Y cells in inflammation.

## 3. Discussion

Iron is an essential trace element necessary for proper brain functioning. Iron homeostasis is tightly regulated by hepcidin—a peptide hormone produced mainly by the liver—but it is also expressed in the brain suggesting its role in the local iron homeostasis [39]. Neuroinflammation is mediated by reactive microglia and astrocytes secreting pro-inflammatory cytokines. These cytokines trigger the production of free radicals, deregulation of iron metabolism and mitochondrial function [40].

Bacterial infections induce cellular responses through Toll-like receptors, a family of pattern recognition receptors [21,22]. Both Gram-positive and Gram-negative bacteria act via these receptors generating immune response in the infected cells, including the cells of the CNS. LTA is a component of the Gram-positive bacterial cell wall, a potent agonist of TLR2 [41]. LPS, from the cell wall of Gram-negative bacteria, is proved to bind to TLR4 and induce activation of microglia [17]. It has been reported that TLR2 and TLR4 are expressed by neurons indicating a role of these receptors in neuronal inflammatory responses [42]. Although the action of microglia and astrocytes under inflammatory conditions is well described, only few articles deal with the behavior of neurons in inflammation. LPS, as an inflammatory mediator has been proved in numerous experiments [43,44] to induce neurodegeneration and dysregulation of iron metabolism, but the direct effect of Gram-positive cell wall component LTA on neuronal iron uptake, storage, and utilization has not been investigated yet.

The aim of our study was to reveal the changes of iron metabolism in differentiated human SH-SY5Y cells under the treatment of inflammatory mediators LPS and LTA, and to examine if the presence of BV-2 murine microglia could alter these changes. To find the answers for these questions we compared the effects of LPS and LTA on monocultured SH-SY5Y cells to BV-2 co-cultured SH-SY5Y cells.

To reveal the alterations in iron metabolism of the LPS and LTA treated monocultures we performed real-time PCR analyses of the genes responsible for iron uptake and storage. Our data strengthens the hypothesis that LPS and LTA can act differently on the iron metabolism of SH-SY5Y cells by changing the expressions of DMT-1 and TfR1—responsible for iron uptake—and FTH and FTMT—responsible for iron storage. The results show that both DMT-1 and TfR1 are involved in iron uptake of LPS- and LTA-treated SH-SY5Y cells but with different degrees, suggesting a feedback mechanism by the iron availability or the iron stores. FTH levels were elevated during LPS while FTMT mRNA levels were increased only in case of LTA treatment of SH-SY5Y cells. These results imply that LPS modifies cytosolic iron stores while LTA influences both the mitochondrial and cytosolic iron stores. Hepcidin secretion and total iron content were significantly higher in the LPS treated SH-SY5Y cells showing a strong correlation among iron uptake, iron storage and hepcidin synthesis.

Our co-culture system is established using human differentiated neuroblastoma cells and murine neonatal microglial cells, the two cell types are able to contact with each other physically by secreting mediators or by releasing extracellular vesicles [45,46] and they react to these stimuli. Moreover, using this co-culture model the cytokine production of the SH-SY5Y cells and BV-2 cells can be examined separately. Previous data have shown that BV-2 cells can be activated by both LPS and LTA which induce the secretion of pro-inflammatory cytokines [8] and the release of NO and glutamate [13,14], and at the presence of pathogens neurons release cytokines to regulate the inflammatory process in the brain [47].

Our results demonstrated that the LPS treated BV-2 cells in the co-culture secrete more IL-6 and TNFα cytokines than LTA treated cells. TNFα secretions of LPS or LTA treated SH-SY5Y cells were altered by BV-2 cells but with different rates. BV-2 cells significantly decreased TNFα secretion of the LPS treated neuronal cells suggesting a protective role of microglia against the TNFα induced neurotoxicity. On the contrary, TNFα production was triggered in LTA treated co-cultured SH-SY5Y cells at 24 h and 48 h compared to the monoculture. However, the BV-2 cells themselves are less sensitive to LTA; they increase the neuronal response to LTA which may influence cell viability (Figure 1).

Previous study reveals that IL-6 and TNFα are also implied in the alteration of iron homeostasis by influencing iron uptake and storage [48]. TNFα has an effect on the iron import on the cells by increasing the expressions of TfR1 and DMT-1 via modulating the activity of IRP1 [49]. Moreover, both TNFα and IL-6 released from SH-SY5Y cells may alter the iron homeostasis by inducing ferritin expression [48]. Based on our data we suggest that the changes in the expression of DMT-1 and TfR1 at the mRNA level are mediated by the neuronal cytokine production. LPS and LTA have different effects on the expression of iron storage genes in monocultured SH-SY5Y cells: upregulated FTH mRNA level in case of LPS treatment, and no remarkable effect in case of LTA treatment. LPS and LTA treatments resulted in elevated intracellular iron content which might trigger FTH expression to decrease iron mediated toxicity and might cause the redistribution of the iron pools. FTMT seems to be affected only by LTA suggesting that LTA increases the mitochondrial iron storage and may affect the reactive nitrogen species production which can influence the functions of the mitochondria (ATP synthesis, iron–sulfur cluster synthesis, and heme synthesis) making the cells more sensitive to LTA.

The protein expression of the examined genes can be modified by inflammatory mediators, by inflammatory cell signaling pathways, iron availability, activity of iron regulatory proteins [48,50,51,52,53], certain miRNAs, as well as by direct contacts with other neuronal cells.

Based on our results we revealed that LPS and LTA act different ways on iron uptake and storage proteins in SH-SY5Y cells and BV-2 cells can modify these protein expression levels. LPS can influence iron uptake by increasing the level of DMT-1 and BV-2 cells can act against LPS by retarding the DMT-1 expression. In case of LTA treatment downregulation of DMT-1 appeared at 24 h in the monocultures while in the co-cultures a decrease in protein level was observed at 48 h suggesting that LTA affects the translational regulation of DMT-1.

The increasing expression of TfR1 at the LPS treated monocultures suggests that TfR1 cooperates with DMT-1 in neuronal iron import. In the co-cultures, BV-2 cells can alter the time of action of LPS on the neuronal expression of TfR1. In case of LTA treatment increased TfR1 protein expression in the monocultures suggests the important role of TfR1 in iron uptake during LTA treatment. In case of LTA treated co-cultures TfR1 level was rather decreased suggesting that DMT-1 is involved in iron uptake at the earlier stage of the treatment then TfR1 may take its role.

The possible reasons for the different actions of LPS and LTA on DMT-1 and TfR1 are the different regulations of iron regulatory proteins (IRPs) [50,51,53]. LPS may contribute to maintain the level of DMT-1 and TfR1 by increasing the activity of IRP1 at the early stage of the treatments [54]. On the contrary, LTA is supposed to decrease the activity of IRP1 contributing to the degradation of DMT-1 mRNA, meanwhile LTA mediated NFκB activation may cause an increment of TfR1 protein level [55]. BV-2 cells may influence also the expression of IRP1 and contribute to the alterations in the expressions of iron uptake proteins. Moreover, elevating level of hepcidin and IL-6 of treated SH-SY5Y cells which both are observed in our experiments, may also contribute to the altered expression of DMT-1 in mono- and co-cultures [35,53]. The discrepancies between protein levels at different time points of the experiments suggest the complex regulation of iron uptake of the SH-SY5Y cells which is influenced by the saturation of iron stores in the cytoplasm and in the mitochondria.

In our experiments FTH protein level was similar to the control level in case of LPS treated monocultures with an exception at 24 h when it decreased. This alteration may be due to the iron redistribution or the increased synthesis of the heme-containing cytoprotective enzymes (e.g., catalases and peroxidases) which protect the cells from iron-mediated ROS production and damage [56]. The BV-2 cells changed this expression pattern by increasing FTH level at LPS treatment of the co-cultures which indicates that LPS treated co-cultured SH-SY5Y cells store more iron in FTH.

LTA treated SH-SY5Y cells showed similar FTH protein expression compared to the controls, while the presence of BV-2 cells it was decreased at 6 h and 48 h and increased at 24 h. This fluctuation is may be caused by the intracellular iron trafficking from the cytosol to the mitochondria and/or by the changes of iron uptake and release. To find out whether the mitochondrial iron store is affected by LPS or LTA we analyzed FTMT protein expression of SH-SY5Y cells. LPS treatment decreased FTMT levels in monocultures. In the LPS-treated co-cultures SH-SY5Y cells had fluctuated FTMT levels. The possible reasons for these results are that SH-SY5Y cells utilize iron for iron–sulfur cluster synthesis and/or heme synthesis or try to export iron from the cytosol [57]. Iron export may initiate iron release from mitochondrion to protect mitochondrial functions from iron mediated stress [58]. It seems that BV-2 cells can trigger downregulation of FTMT earlier, but feedback mechanisms may act against this effect and increases FTMT protein level later. On the contrary, overproduction of FTMT can cause cytosolic iron deficiency with low ferritin and high TfR1 levels [59]. This modulatory effect of FTMT was observed in the LTA treated co-cultures at 48 h. The presence of microglial cells alters the iron storage conditions: at 24 h FTH is increased, at 48 h FTH was decreased and FTMT expression was increased suggesting iron transport between cytosol and mitochondria mediated by BV-2 cells. Increased FTMT levels are supposed to protect the cells from increasing labile iron pool and iron mediated oxidative damage [60], iron overload in the mitochondrion may interfere with the normal mitochondrial functions and lead to cell death [61]. Since FTMT lacks functional IRE and its expression is not regulated by IRPs, other regulatory mechanisms might be the underlying causes of the expressional alterations contributing iron redistribution. The action of miRNAs on mRNA molecules decreases the translation of the genes which are involved in iron metabolism [62]. miRNA expression depends on the dietary iron intake [63] thus they may have a crucial role in the alterations of DMT-1, TfR1, FTH, and FTMT. The mRNAs transcribed from the first three genes are regulated directly by miRNAs [64], while FTMT mRNA is regulated indirectly through the control of iron–sulfur biogenesis [65].

The role of hepcidin in maintaining and regulating the brain iron homeostasis is still emerging. It has been shown that inflammatory stimuli did not trigger the expression of hepcidin in neurons [52] while other authors revealed that LPS upregulated hepcidin in neurons via the IL-6/STAT3 pathway [66]. In our study it was proved that BV-2 cells had an opposite effect on hepcidin secretion in LPS and LTA treated co-cultures. BV-2 cells seemed to attenuate the effect of LPS on SH-SY5Y cells by decreasing significantly the hepcidin production of SH-SY5Y cells, meanwhile BV-2 cells increased significantly the hepcidin secretion in case of LTA treated SH-SY5Y cells. The changes in the secretion of hepcidin in SH-SY5Y cells are supposed to contribute to the alterations of the iron uptake and storage of neurons. Our data also demonstrated that both LPS and LTA increased IL-6 production of SH-SY5Y cells and phosphorylation of STAT3 protein, which was correlated with increased hepcidin production in LPS treated mono- and co-cultures, although it seemed that BV-2 cells caused a delay in STAT3 phosphorylation.

The alterations in the expression of iron uptake and storage proteins and hepcidin secretion in SH-SY5Y cells presumed increased cellular iron content, although these changes were found to be diverse in the mono- and co-cultures. Moreover, LTA treated co-cultured SH-SY5Y cells augmented their iron content more than the LPS-treated cells. Although it is supposed that microglia provide a protection to the neurons against iron overload [67], our results demonstrated that BV-2 microglia triggered the neurons to increase their iron content. In addition, based on our measurements, the BV-2 cells contributed to the increased iron accumulation of SH-SY5Y cells by releasing their iron content into the culture media and probably enhancing the uptake of non-transferrin-bound iron by neurons. The hepcidin secretion of the SH-SY5Y cells correlated with the increasing iron content indicating that hepcidin acts on the neurons in an autocrine way and inhibits the release of iron from the cell.

Although the role of microglia in inflammatory processes and neurodegeneration has been intensively investigated, the reaction of neurons to inflammatory stimulus is not well understood [68]. Emerging evidences strengthen the importance of the investigations on neurons to reveal which intracellular mechanisms become active or inactive during neuroinflammation and how these mechanisms are altered by microglia, the major regulators of inflammatory processes of the brain. Unraveling the molecular mechanisms involved in the changes of neural metabolism at inflammation may help to elucidate the pathophysiological processes contributing to iron accumulation and cell death.

In summary, our study strengthens the role of inflammation as an underlying cause of iron accumulation in the neurons. Although many regulatory partners and direct or indirect cellular interactions remained to be further examined, we revealed that microglia contributed to the increased iron content of the neurons suggesting their role in neuronal iron overload due to neuroinflammation. Though LPS and LTA both activate the NFκB pathway, MAP kinase cascades, and the secretion of pro-inflammatory cytokines, our results revealed that they triggered different responses in the differentiated SH-SY5Y cells, although it seems that LPS is a stronger influencing factor for the regulation neuronal iron metabolism. BV-2 microglial cells influenced the effects of LPS and LTA by changing iron uptake and storage conditions, and cytokine secretions of the neurons. Taken together we suggest that differentiated SH-SY5Y cells are able to react differently to the presence of LPS and LTA and that their reactions are significantly modulated by the microglia proving that microglia are important regulators of neuronal iron metabolism and neuronal survival.

## 4. Materials and Methods

### 4.1. Cell Cultures and Treatments

SH-SY5Y neuroblastoma cells (ATCC, CRL-2266) were cultured in DMEM/F12 medium (Lonza Ltd. Basel, Switzerland) and supplemented with 10% fetal bovine serum (FBS, EuroClone S.p.A, Pero, Italy) and 1% nonessential amino acids (NEAA, Lonza) and 1% Penicillin–Streptomycin (P/S, Lonza). Cells (1 × 10^6^) were seeded onto 25 cm^3^ flasks in DMEM/F12 medium supplemented with 1% FBS and 1% NEAA and were differentiated with 1 µM all-trans retinoic acid (ATRA, Sigma-Aldrich Kft, Budapest, Hungary) for 5 days in a humidified atmosphere containing 5% CO_2_ at 37 °C. For the co-culture experiments SH-SY5Y cells were seeded and differentiated on Thermanox coverslips (Thermo Fisher Scientific Inc., Waltham, MA, USA) in 6-well dishes (TPP Techno Plastic Products AG, Trasadingen, Switzerland). BV-2 murine microglial cells (a kind gift from Prof. László Tretter and his research group) were maintained in Dulbecco’s Modified Eagle’s Medium (DMEM) (Lonza) supplemented with 10% FBS and 1% P/S. The cells were plated on poly-l-ornithine (Sigma-Aldrich Kft.) coated dishes (Corning Inc., Corning, NY, USA) and after 24 h differentiated SH-SY5Y cells were added to the BV-2 cells by turning the coverslips upside down with SH-SY5Y cells facing the microglial cells, so the cells are separated by a thin layer of culture medium. Bilaminar co-cultures were supplemented with 10 µM of cytosine-β-d-arabinofuranoside (Sigma-Aldrich Kft.), in order to prevent glia proliferation and migration [69]. LPS (*E. coli* 055:B5, Sigma-Aldrich Kft.) and LTA (*Staphylococcus aureus*, Sigma-Aldrich Kft.) were used in the same concentrations (1 µg/mL) in all experiments. Mono- and co-cultured SH-SY5Y cells were treated with LPS or LTA separately. Cell cultures were treated for 6 h, 24 h, and 48 h to reveal the early and late changes in mRNA expressions. Untreated cells were used as controls.

### 4.2. Real-Time PCR

SH-SY5Y cells in the monoculture were harvested after washing with PBS Sigma-Aldrich Kft.). Total RNA was isolated from each sample using Quick RNA mini kit (Zymo Research, Irvine, CA, USA). Complementary DNA was synthesized from 200 ng total RNA using a high-capacity cDNA reverse transcription kit (Thermo Fisher Scientific Inc.) according to the manufacturer’s protocol. Determination of gene expressions was performed in a CFX96 Real-time System (Bio-Rad Inc. Hercules, CA, USA) using iTaq™ Universal SYBR^®^ Green Supermix (Bio-Rad Inc.) in a total reaction volume of 20 µL. Melting curves were generated after each quantitative PCR run to ensure that a single specific product was amplified. Both target and reference genes were amplified with efficiencies near 100%. Relative quantification was calculated by the ∆∆Ct (Livak) method using the Bio-Rad CFX Manager 3.1 software (Bio-Rad Inc.). The expression level of the gene of interest was compared with the level of β-actin in each sample. These relative expression rates were then compared between the treated and untreated samples. Relative expression of controls was set to 1. Untreated cell controls were made at each examined time point of the treatments, 6 h, 24 h, and 48 h, respectively. The mRNA expressions of the treated cells were compared to the appropriate controls. Primers are described in Table 1.

### 4.3. Western Blotting

SH-SY5Y cells in the monoculture were harvested after washing with phosphate buffer saline (PBS) (Sigma-Aldrich Kft.). The co-cultured SH-SY5Y cells were separated from BV-2 cells by removing the coverslips from the surface of BV-2 cells. The coverslips were washed with PBS (Sigma-Aldrich Kft.) and the cells were collected by trypsinization. Pelleted cells were lysed with 130 µL of M-PER Mammalian Protein Extraction Reagent (Thermo Fisher Scientific Inc.) supplemented with complete mini protease inhibitor cocktail (Roche Ltd., Basel, Switzerland). Protein contents of the samples were measured with DC Protein Assay Kit (Bio-Rad Inc.). The same amount of protein (15 µg) from each sample was separated by sodium dodecyl sulfate–polyacrylamide gel electrophoresis (SDS-PAGE) using a 12% or 14% polyacrylamide gel and transferred by electroblotting to nitrocellulose membranes. The membranes were probed with the following polyclonal rabbit antibodies according to the manufacturers’ protocol: anti-DMT-1 Immunoglobulin G (IgG) (1:1000; Novus Biologicals, Bio-Techne Corporation, Cambridge, UK), anti-TfR1 IgG (1:1000; Thermo Fisher Scientific Inc.), anti-FTMT IgG (1:1000; Thermo Fisher Scientific Inc.), anti-FTH IgG (1:1000; Cell Signaling Technology Europe, Leiden, The Netherlands), and antiphosphorylated signal transducer and activator of transcription 3 (p-STAT3) IgG (1:2000; Cell Signaling Technology Europe). β-actin (1:2000; Sigma-Aldrich Kft.) was used as loading control.

### 4.4. Enzyme-Linked Immunosorbent Assay (ELISA) Measurements

After each treatment, culture media of treated and control cells were collected and stored at −80 °C until the measurements. The mature hepcidin content of each sample was determined with Hepcidin 25 bioactive ELISA Kit (DRG Diagnostics GmbH, Marburg, Germany) according to the manufacturer’s protocol. Secreted IL-6 and TNFα concentrations of the culture media of SH-SY5Y monocultures and co-cultures were determined with human and mouse IL-6 and human and mouse TNFα ELISA Kits (Thermo Fisher Scientific Inc.), respectively, according to the instructions of the manufacturer. The hepcidin and cytokine contents of the control media did not change during the experiments therefore only one control was represented in the figures.

### 4.5. Determination of the Total Iron Content of Cultured Cells

Determination of the iron concentration was performed using a colorimetric ferrozine-based assay described by Riemer et al. [70]. Briefly the cells were collected and lysed with 50 mM NaOH at room temperature for 2 h. After the incubation the samples were mixed with iron releasing reagent (1.4 M HCl, 4.5% (wt/vol) KMnO_4_ in H_2_O) and were incubated for 2 h at 60 °C then iron detection reagent (6.5 mM ferrozine; 6.5 mM neocuproine; 2.5 M ammonium acetate; 1 M ascorbic acid) was added to each tube and incubated at RT for 30 min. The absorbance was measured at 492 nm on EnSpire Multimode plate reader (PerkinElmer, Rodgau, Germany). The concentration was determined by a standard curve using FeCl_3_ (0–300 µM) treated the same way as the samples. Protein concentration was measured from each sample with DC Protein Assay Kit (Bio-Rad Inc.). Iron content was normalized against the protein content and was expressed as µM iron/mg protein. Iron contents of the untreated control cells were measured the same way and at the same time points of the experiments (6 h, 24 h, and 48 h).

### 4.6. Statistical Analysis

The data presented are representative of at least three independent experiments. For all data, *n* corresponds to the number of independent experiments. Statistical analysis was performed using SPSS software (IBM Corporation, Armonk, NY, USA). Statistical significance was determined using Student’s *t*-test to compare the two treated groups and to compare controls to treated groups. We used Bonferroni correction to adjust probability (*p*)-values because of the increased risk of a type I error when making multiple statistical tests. Values were expressed as mean ± standard errors of the mean (SEM). Statistical significance was set at *p*-value < 0.01.

## Figures and Tables

**Table 1:** Real-time PCR gene primer list.

Primer	Sequence 5′ → 3′
**DMT-1 forward**	GTGGTTACTGGGCTGCATCT
**DMT-1 reverse**	CCCACAGAGGAATTCTTCCT
**TfR1 forward**	CATGTGGAGATGAAACTTGC
**TfR1 reverse**	TCCCATAGCAGATACTTCCA
**FTH forward**	GAGGTGGCCGAATCTTCCTTC
**FTH reverse**	TCAGTGGCCAGTTTGTGCAG
**FTMT forward**	AAGGTGACCCCCATTTGTGC
**FTMT reverse**	GGGGCCCCCATCTTCACTAA
**β-actin forward**	AGAAAATCTGGCACCACACC
**β-actin reverse**	GGGGTGTTGAAGGTCTCAAA

## Supplementary Materials

Figure S1: Optical densities of the western blot analyses of DMT-1 (A,B), TfR1 (C,D), FTH (E,F), and FTMT (G,H) of mono- and co-cultured SH-SY5Y cells. The analyses were made using ImageJ software (https://imagej.nih.gov/ij/), the optical density of the examined proteins was expressed as percentage of target protein/β-actin abundance. Asterisk marks *p* < 0.01 compared to untreated controls. Figure S2: Optical densities of the Western blot analyses of p-STAT3 of mono- (A) and co-cultured (B) SH-SY5Y cells. The analyses were made using ImageJ software (https://imagej.nih.gov/ij/); the optical density of p-STAT3 was expressed as percentage of target protein/β-actin abundance. Asterisk marks *p* < 0.01 compared to untreated controls.

## References

[1] Medzhitov R. (2007). TLR-mediated innate immune recognition. Semin. Immunol..

[2] Dal-Pizzol F., Tomasi C.D., Ritter C. (2014). Septic encephalopathy: DOES inflammation drive the brain crazy?. Rev. Bras. Psiquiatr..

[3] Michels M., Sonai B., Dal-Pizzol F. (2017). Polarization of microglia and its role in bacterial sepsis. J. Neuroimmunol..

[4] Da Fonseca A.C., Matias D., Garcia C., Amaral R., Geraldo L.H., Freitas C., Lima F.R. (2014). The impact of microglial activation on blood-brain barrier in brain diseases. Front. Cell. Neurosci..

[5] Sonneville R., Verdonk F., Rauturier C., Klein I.F., Wolff M., Annane D., Chretien F., Sharshar T. (2013). Understanding brain dysfunction in sepsis. Ann. Intensive Care.

[6] Liu Y.C., Zou X.B., Chai Y.F., Yao Y.M. (2014). Macrophage polarization in inflammatory diseases. Int. J. Biol. Sci..

[7] Michels M., Danielski L.G., Dal-Pizzol F., Petronilho F. (2014). Neuroinflammation: MICROGLIAL activation during sepsis. Curr. Neurovasc. Res..

[8] Park S.Y., Kim Y.H., Park G. (2016). Anti-neuro-inflammatory effects of Nardostachys chinensis in lipopolysaccharide-and lipoteichoic acid-stimulated microglial cells. Chin. J. Nat. Med..

[9] Danielski L.G., Giustina A.D., Badawy M., Barichello T., Quevedo J., Dal-Pizzol F., Petronilho F. (2018). Brain Barrier Breakdown as a Cause and Consequence of Neuroinflammation in Sepsis. Mol. Neurobiol..

[10] Rahman O., Dover L.G., Sutcliffe I.C. (2009). Lipoteichoic acid biosynthesis: TWO steps forwards, one step sideways?. Trends Microbiol..

[11] Ray A., Cot M., Puzo G., Gilleron M., Nigou J. (2013). Bacterial cell-wall macroamphiphiles: PATHOGEN-/microbe-associated molecular patterns detected by mammalian innate immune system. Biochimie.

[12] Baik J.E., Jang K.S., Kang S.S., Yun C.H., Lee K., Kim B.G., Kum K.Y., Han S.H. (2011). Calcium hydroxide inactivates lipoteichoic acid from Enterococcus faecalis through deacylation of the lipid moiety. J. Endod..

[13] Han S.H., Kim J.H., Seo H.S., Martin M.H., Chung G.H., Michalek S.M., Nahm M.H. (2006). Lipoteichoic acid-induced nitric oxide production depends on the activation of platelet-activating factor receptor and Jak2. J. Immunol..

[14] Brown G.C., Neher J.J. (2010). Inflammatory neurodegeneration and mechanisms of microglial killing of neurons. Mol. Neurobiol..

[15] Heneka M.T., Feinstein D.L. (2011). Expression and function of inducible nitric oxide synthase in neurons. J. Neuroimmunol..

[16] Asehnoune K., Strassheim D., Mitra S., Kim J.Y., Abraham E. (2004). Involvement of reactive oxygen species in Toll-like receptor 4-dependent activation of NF-kappa B. J. Immunol..

[17] De Nardo D. (2015). Toll-like receptors: Activation, signalling and transcriptional modulation. Cytokine.

[18] Kopp E., Medzhitov R. (2003). Recognition of microbial infection by Toll-like receptors. Curr. Opin. Immunol..

[19] Gay N.J., Gangloff M. (2007). Structure and function of Toll receptors and their ligands. Annu. Rev. Biochem..

[20] Tapping R.I., Akashi S., Miyake K., Godowski P.J., Tobias P.S. (2000). Toll-like receptor 4, but not toll-like receptor 2, is a signaling receptor for Escherichia and Salmonella lipopolysaccharides. J. Immunol..

[21] Lien E., Ingalls R.R. (2002). Toll-like receptors. Crit. Care Med..

[22] Palliser D., Huang Q., Hacohen N., Lamontagne S.P., Guillen E., Young R.A., Eisen H.N. (2004). A role for Toll-like receptor 4 in dendritic cell activation and cytolytic CD8+ T cell differentiation in response to a recombinant heat shock fusion protein. J. Immunol..

[23] Rosenberger K., Dembny P., Derkow K., Engel O., Kruger C., Wolf S.A., Kettenmann H., Schott E., Meisel A., Lehnardt S. (2015). Intrathecal heat shock protein 60 mediates neurodegeneration and demyelination in the CNS through a TLR4- and MyD88-dependent pathway. Mol. Neurodegener..

[24] Tu Z., Portillo J.A., Howell S., Bu H., Subauste C.S., Al-Ubaidi M.R., Pearlman E., Lin F. (2011). Photoreceptor cells constitutively express functional TLR4. J. Neuroimmunol..

[25] Yang N.J., Chiu I.M. (2017). Bacterial Signaling to the Nervous System through Toxins and Metabolites. J. Mol. Biol..

[26] Crichton R.R., Dexter D.T., Ward R.J. (2011). Brain iron metabolism and its perturbation in neurological diseases. J. Neural Transm..

[27] Rouault T.A., Cooperman S. (2006). Brain iron metabolism. Semin. Pediatr. Neurol..

[28] Biasiotto G., Di Lorenzo D., Archetti S., Zanella I. (2016). Iron and Neurodegeneration: Is Ferritinophagy the Link?. Mol. Neurobiol..

[29] Gao G., Chang Y.Z. (2014). Mitochondrial ferritin in the regulation of brain iron homeostasis and neurodegenerative diseases. Front. Pharmacol..

[30] Bishop G.M., Dang T.N., Dringen R., Robinson S.R. (2011). Accumulation of non-transferrin-bound iron by neurons, astrocytes, and microglia. Neurotox. Res..

[31] Mena N.P., Bulteau A.L., Salazar J., Hirsch E.C., Nunez M.T. (2011). Effect of mitochondrial complex I inhibition on Fe-S cluster protein activity. Biochem. Biophys. Res. Commun..

[32] Park C.H., Valore E.V., Waring A.J., Ganz T. (2001). Hepcidin, a urinary antimicrobial peptide synthesized in the liver. J. Biol. Chem..

[33] Nemeth E., Tuttle M.S., Powelson J., Vaughn M.B., Donovan A., Ward D.M., Ganz T., Kaplan J. (2004). Hepcidin regulates cellular iron efflux by binding to ferroportin and inducing its internalization. Science.

[34] Wang J., Pantopoulos K. (2011). Regulation of cellular iron metabolism. J. Biochem..

[35] Urrutia P., Aguirre P., Esparza A., Tapia V., Mena N.P., Arredondo M., Gonzalez-Billault C., Nunez M.T. (2013). Inflammation alters the expression of DMT1, FPN1 and hepcidin, and it causes iron accumulation in central nervous system cells. J. Neurochem..

[36] Andersen H.H., Johnsen K.B., Moos T. (2014). Iron deposits in the chronically inflamed central nervous system and contributes to neurodegeneration. Cell. Mol. Life Sci..

[37] Stansley B., Post J., Hensley K. (2012). A comparative review of cell culture systems for the study of microglial biology in Alzheimer’s disease. J. Neuroinflamm..

[38] Szutowicz A., Bielarczyk H., Jankowska-Kulawy A., Ronowska A., Pawelczyk T. (2015). Retinoic acid as a therapeutic option in Alzheimer’s disease: A focus on cholinergic restoration. Expert Rev. Neurother..

[39] Zechel S., Huber-Wittmer K., von Bohlen und Halbach O. (2006). Distribution of the iron-regulating protein hepcidin in the murine central nervous system. J. Neurosci. Res..

[40] Urrutia P.J., Mena N.P., Nunez M.T. (2014). The interplay between iron accumulation, mitochondrial dysfunction, and inflammation during the execution step of neurodegenerative disorders. Front. Pharmacol..

[41] Mitchell L., Smith S.H., Braun J.S., Herzog K.H., Weber J.R., Tuomanen E.I. (2004). Dual phases of apoptosis in pneumococcal meningitis. J. Infect. Dis..

[42] Dzamko N., Gysbers A., Perera G., Bahar A., Shankar A., Gao J., Fu Y., Halliday G.M. (2017). Toll-like receptor 2 is increased in neurons in Parkinson’s disease brain and may contribute to alpha-synuclein pathology. Acta Neuropathol..

[43] Neher J.J., Brown G.C. (2007). Neurodegeneration in models of Gram-positive bacterial infections of the central nervous system. Biochem. Soc. Trans..

[44] Kinsner A., Pilotto V., Deininger S., Brown G.C., Coecke S., Hartung T., Bal-Price A. (2005). Inflammatory neurodegeneration induced by lipoteichoic acid from Staphylococcus aureus is mediated by glia activation, nitrosative and oxidative stress, and caspase activation. J. Neurochem..

[45] Bureau G., Longpre F., Martinoli M.G. (2008). Resveratrol and quercetin, two natural polyphenols, reduce apoptotic neuronal cell death induced by neuroinflammation. J. Neurosci. Res..

[46] Cook A., Hippensteel R., Shimizu S., Nicolai J., Fatatis A., Meucci O. (2010). Interactions between chemokines: REGULATION of fractalkine/CX3CL1 homeostasis by SDF/CXCL12 in cortical neurons. J. Biol. Chem..

[47] Benveniste E.N. (1992). Inflammatory cytokines within the central nervous system: SOURCES, function, and mechanism of action. Am. J. Physiol..

[48] Torti F.M., Torti S.V. (2002). Regulation of ferritin genes and protein. Blood.

[49] Wang J., Song N., Jiang H., Wang J., Xie J. (2013). Pro-inflammatory cytokines modulate iron regulatory protein 1 expression and iron transportation through reactive oxygen/nitrogen species production in ventral mesencephalic neurons. Biochim. Biophys. Acta.

[50] Henderson B.R. (1996). Iron regulatory proteins 1 and 2. BioEssays.

[51] Cairo G., Recalcati S. (2007). Iron-regulatory proteins: MOLECULAR biology and pathophysiological implications. Expert Rev. Mol. Med..

[52] Finazzi D., Arosio P. (2014). Biology of ferritin in mammals: AN update on iron storage, oxidative damage and neurodegeneration. Arch. Toxicol..

[53] Kuhn L.C. (2015). Iron regulatory proteins and their role in controlling iron metabolism. Metallomics.

[54] Recalcati S., Pometta R., Levi S., Conte D., Cairo G. (1998). Response of monocyte iron regulatory protein activity to inflammation: ABNORMAL behavior in genetic hemochromatosis. Blood.

[55] Li L., Holscher C., Chen B.B., Zhang Z.F., Liu Y.Z. (2011). Hepcidin treatment modulates the expression of divalent metal transporter-1, ceruloplasmin, and ferroportin-1 in the rat cerebral cortex and hippocampus. Biol. Trace Elem. Res..

[56] Arosio P., Levi S. (2010). Cytosolic and mitochondrial ferritins in the regulation of cellular iron homeostasis and oxidative damage. Biochim. Biophys. Acta.

[57] Lv H., Shang P. (2018). The significance, trafficking and determination of labile iron in cytosol, mitochondria and lysosomes. Metallomics.

[58] Mühlenhoff U., Hoffmann B., Richter N., Rietzschel N., Spantgar F., Stehling O., Uzarska A.M., Lill R. (2015). Compartmentalization of iron between mitochondria and the cytosol and its regulation. Eur. J. Cell Biol..

[59] Nie G., Sheftel A.D., Kim S.F., Ponka P. (2005). Overexpression of mitochondrial ferritin causes cytosolic iron depletion and changes cellular iron homeostasis. Blood.

[60] Guan H., Yang H., Yang M., Yanagisawa D., Bellier J.P., Mori M., Takahata S., Nonaka T., Zhao S., Tooyama I. (2017). Mitochondrial ferritin protects SH-SY5Y cells against H2O2-induced oxidative stress and modulates alpha-synuclein expression. Exp. Neurol..

[61] Huang H., Chen J., Lu H., Zhou M., Chai Z., Hu Y. (2017). Iron-induced generation of mitochondrial ROS depends on AMPK activity. Biometals.

[62] Hamara K., Bielecka-Kowalska A., Przybylowska-Sygut K., Sygut A., Dziki A., Szemraj J. (2013). Alterations in expression profile of iron-related genes in colorectal cancer. Mol. Biol. Rep..

[63] Davis M., Clarke S. (2013). Influence of microRNA on the maintenance of human iron metabolism. Nutrients.

[64] Xu Z., Shi Z., Li Y. (2014). The Crosstalk between Micro RNA and Iron Homeostasis. Int. J. Genom. Med..

[65] Yoshioka Y., Kosaka N., Ochiya T., Kato T. (2012). Micromanaging Iron Homeostasis: HYPOXIA-inducible micro-RNA-210 suppresses iron homeostasis-related proteins. J. Biol. Chem..

[66] Qian Z.M., He X., Liang T., Wu K.C., Yan Y.C., Lu L.N., Yang G., Luo Q.Q., Yung W.H., Ke Y. (2014). Lipopolysaccharides upregulate hepcidin in neuron via microglia and the IL-6/STAT3 signaling pathway. Mol. Neurobiol..

[67] Thomsen M.S., Andersen M.V., Christoffersen P.R., Jensen M.D., Lichota J., Moos T. (2015). Neurodegeneration with inflammation is accompanied by accumulation of iron and ferritin in microglia and neurons. Neurobiol. Dis..

[68] Ramesh G., Philipp M.T., Vallieres L., MacLean A.G., Ahmad M. (2013). Mediators of neuroinflammation. Mediat. Inflamm..

[69] Shimizu S., Abt A., Meucci O. (2011). Bilaminar co-culture of primary rat cortical neurons and glia. J. Vis Exp..

[70] Riemer J., Hoepken H.H., Czerwinska H., Robinson S.R., Dringen R. (2004). Colorimetric ferrozine-based assay for the quantitation of iron in cultured cells. Anal. Biochem..

