# Supplementary figures and images for: Effect of Inflammatory Mediators Lipopolysaccharide and Lipoteichoic Acid on Iron Metabolism of Differentiated SH-SY5Y Cells Alters in the Presence of BV-2 Microglia
Source: Int J Mol Sci. 2018 Dec 20;20(1):17. doi: 10.3390/ijms20010017 (PMC6337407; doi:10.3390/ijms20010017)

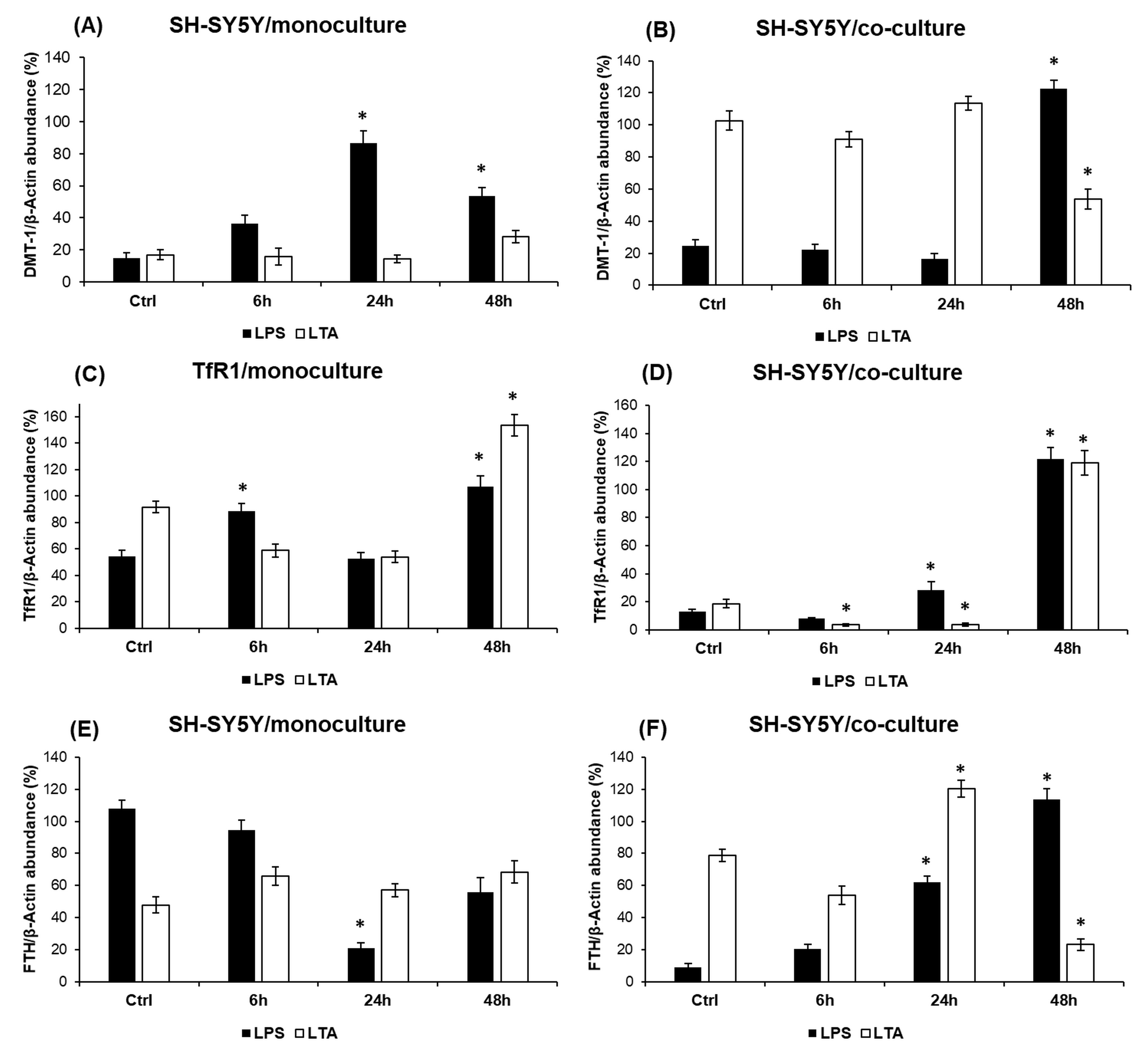

Supplement: Supplementary file 1 [file ijms-20-00017-s001.zip › Figure S1.tiff]

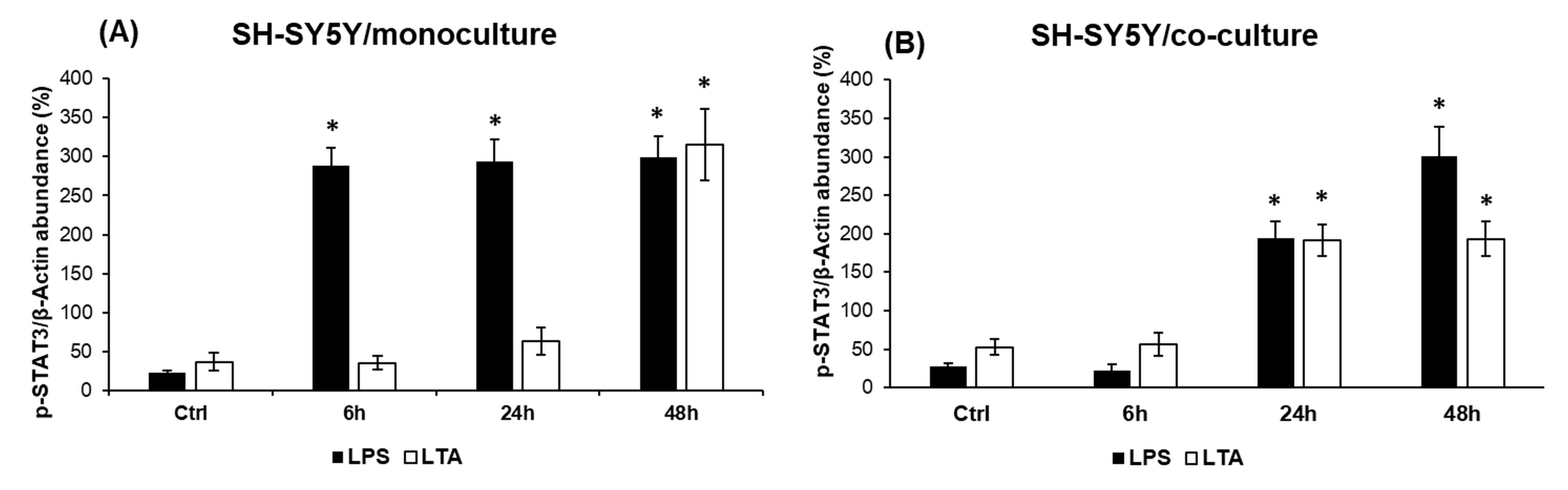

Supplement: Supplementary file 1 [file ijms-20-00017-s001.zip › Figure S2.tiff]
